# Promoting and sustaining fruit intake among children aged 3–11 years: a before-and-after evaluation of a school-based intervention

**DOI:** 10.1007/s00394-026-04042-3

**Published:** 2026-06-29

**Authors:** Raquel Martins, João Fernandes, Rodrigo Feteira-Santos, Ana Virgolino, Rita Santos Loureiro, Mário Silva, José Camolas, Osvaldo Santos

**Affiliations:** 1 Environmental Health Behaviour Lab, Institute of Environmental Health, School of Medicine, University of Lisbon, Lisbon, Portugal; 2 Childhood Obesity Association, Armação de Pêra, Portugal; 3 Unbreakable Idea Research, Cadaval, Portugal

**Keywords:** Child, Health promotion, Early intervention education, Habits, Gamification

## Abstract

**Purpose:**

Insufficient fruit intake remains a public health concern in childhood, with potential long-term health impacts. This study assessed the adequacy of a school-based intervention in promoting and sustaining fruit intake among Portuguese children aged 3–11 years.

**Methods:**

A before-and-after study was conducted in a non-probabilistic sample of pre- and primary schools across Portugal. Children participated in 12-week teacher-led classroom activities encouraging fruit consumption using storytelling and gamification strategies. Fruit intake was assessed by students’ daily self-reports and teachers’ records of portions eaten the previous day, collected at baseline and post-intervention. Regular fruit intake was defined as eating fruit on at least 75% of school days for four consecutive weeks, sustained through completion. A *per-protocol* approach was applied, with behavioral analysis restricted to children consuming < 3 portions/day at baseline. Pre- and post-intervention differences were assessed using paired Student’s t-tests. Kaplan–Meier survival analysis was used to evaluate the timing of regular fruit intake enactment, accounting for the design effect.

**Results:**

Among 1016 children (64 schools) with complete pre- and post-intervention data, 357 provided daily records and had suboptimal baseline intake. Over half achieved regular fruit intake at school by week 12, with most gains observed by week 4. Mean overall fruit intake increased from 2.09 (1.12) to 2.44 (1.02) portions/day (*p* < 0.001). Children previously exposed to the intervention were 2.24 times more likely to meet fruit recommendations at baseline.

**Conclusion:**

Findings suggest improvements in fruit intake following this school-based intervention, and that repeated exposure may help consolidate healthy eating habits.

**Supplementary Information:**

The online version contains supplementary material available at 10.1007/s00394-026-04042-3.

## Introduction

Early-life eating behaviors often persist into adulthood and may influence long-term health [1–3]. Evidence from longitudinal trials has shown that higher fruit and vegetable intake during childhood is linked with a reduced risk of non-communicable diseases in adulthood [4, 5]. Moreover, individuals with greater vegetable consumption early in life show lower rates of cardiovascular-related mortality [5]. Therefore, encouraging healthy eating behaviors among children represents a potential strategy to mitigate the burden of diet-related outcomes.

The updated World Health Organization guidelines set age-specific minimum daily intakes for fruit and vegetables: 250 g for children aged 2–5 years, 350 g for ages 6–9, and 400 g for those aged 10 and older [6]. In Portugal, the Food Wheel Guide advises 3–5 portions of fruit per day (one portion corresponds to 160 g), being the lower limit intended for children; [7]. Nevertheless, fruit intake in Portugal remains suboptimal. According to the National Food, Nutrition and Physical Activity Survey 2015–2016, children aged 3–9 years consumed, on average, 109 g of fresh fruit per day [8]. Among Portuguese 11-year-olds, previous studies reported mean daily intakes of 153 g in 2003 [9] and 137 g in 2009 [10]. National surveillance data among children aged 6–8 years showed that the proportion reporting daily fresh fruit intake (overall, including at home and at school) increased from 63.1% in 2019 [11] to 71.2% in 2021 [12]. However, these data reflect consumption frequency rather than quantitative intake, limiting direct assessment of compliance with current fruit intake recommendations.

Several authors have explored interventions promoting fruit or fruit-and-vegetable intake among children across different settings. Overall, school-based initiatives have modest short-term improvements, particularly among children aged 3–6 years [13] and 6–12 years [14, 15]. A meta-analysis examining long-term outcomes (1–24 months post-intervention) among children between 5 and 12 years old estimated a significant increase of 0.24 daily fruit portions in the intervention group compared to controls [16]. 

*‘Fruit Heroes*^®^– *Healthy School Snacks’* is the largest school-based initiative in Portugal promoting healthy eating – specifically fruit intake –, developed and implemented by the Portuguese Childhood Obesity Association. Targeting children aged 3–11 years, the intervention was set to run for 12 consecutive weeks and encourages daily fruit intake through a motivational approach that combines gamification and storytelling techniques [17].

The formation of health-related habits, such as fruit intake, is a gradual process that requires repeated practice in the same context until the behavior becomes automatic. With repetition, control progressively shifts from deliberated intention to automatic responses triggered by environmental cues. According to Lally and colleagues [18], this process takes, on average, 12 weeks (for daily performed behavior), which informed the decision to structure the ‘*Fruit Heroes*^®’^ with this duration.

Since its launch in 2011, the intervention has been implemented free of charge in schools nationwide, reaching over one million children. Given its wide reach, sustained delivery, and potential for replication, ‘*Fruit Heroes*^®’^ may serve as a model for public health interventions in schools. Its formal evaluation is needed to generate evidence that can inform future strategies to improve dietary behaviors in childhood. This was the motivation for this study, which aimed to assess the adequacy of the ‘*Fruit Heroes*^®’^ intervention in increasing overall fruit intake and promoting its regular consumption at school among Portuguese children aged 3–11 years (pre- and primary school).

In the present study, *adequacy* is understood as the extent to which the intervention activities have met the intended objectives, assessed through changes in relevant behavioral indicators (i.e., the increase and maintenance of fruit intake) over time [19].

## Materials and methods

This study followed a non-controlled, community-based trial design with before-and-after assessments. The intervention was implemented in pre- and primary schools across all regions of Portugal (including Azores and Madeira Autonomous Regions).

### Description of the intervention

‘*Fruit Heroes*^®’^ is a 12-week school-based intervention combining educational materials with non-formal learning strategies to promote daily fruit intake. Fruit is brought to school either by parents as part of daily school snacks or, in some municipalities, through local initiatives that support the provision of fruit for free in schools as part of broader health promotion initiatives. The intervention follows the “3R method”: role modeling, repetition, reinforcement. Role modelling was applied in daily 15-minute classroom sessions, during which teachers read or played an audio version of a playful-pedagogic tale featuring the *Fruit Heroes*—a group of characters who gain “superpowers” (i.e., health benefits) by consuming fruit. This storytelling component aimed to foster students’ engagement with the characters and encourage them to associate fruit with positive health outcomes. Repetition was encouraged through gamification using a classroom tracking chart, where children earned points every day they ate fruit at school. To further strengthen behavior change, the intervention incorporates reinforcement strategies. Children received weekly incentives (e.g., bracelets) to motivate them to repeat the behavior until it becomes a habit. All participating teachers received online training before implementation to ensure familiarity with the intervention materials and to standardize the delivery of activities and data collection.

### Sampling

During the 2018–2019 school year, the Childhood Obesity Association in Portugal invited all public and private pre- and primary schools nationwide (over 4,000 institutions) to participate in the ‘*Fruit Heroes*^®’^ intervention (census approach), by email or telephone. Within each school that agreed to participate (*n* = 625), all teachers were invited to take part in the study. Participants were enrolled after informed consent was obtained from their parents/legal guardians and the child expressed agreement.

A non-probabilistic subsample of classrooms (*n* = 93) was selected from those who agreed to participate, being asked to report daily fruit intake throughout the 12 weeks. These schools represented all main Portuguese regions (NUTS II) [20]. The number of classrooms was proportional to overall regional participation.

### Data collection instruments and procedures

Data were collected between October 2018 and February 2019 through self-administered instruments completed by students and teachers.

#### Sample characterization

Teachers collected sociodemographic data at baseline, including a unique child identification code (ensuring data confidentiality and longitudinal tracking), school, class, sex, date of birth, and participation status (first-time or previous exposure to ‘*Fruit Heroes*^®’^). For analysis purposes, age was grouped into 3–5 years, 6–8 years, and 9–11 years.

#### Fruit intake

This outcome was assessed using two complementary measures.

Overall fruit intake was recorded by the number of fruit portions consumed on the previous day, registered by the teacher based on the child’s report at two timepoints: before and after the intervention. To standardize quantification, each teacher received a list of fruit equivalents specifying the quantity of each fruit corresponding to one portion. Based on the Portuguese Food Wheel Guide [7], daily intake was recoded as below (< 3 portions/day), within (3–5 portions/day), or above (> 5 portions/day) recommendations.

Additionally, regular fruit intake at school was assessed using a classroom tracking chart, completed daily by children under teacher supervision, indicating whether fruit had been consumed during morning or afternoon school snacks (yes/no format). This activity was carried out during the first and last week of the intervention for the total sample, and continuously throughout the 12 weeks for the subsample. Although students were identified on the chart, only anonymized data were included in the database – linked to the child’s identification code (student’s number assigned alphabetically by schools), class, and school. The same ID was used across all instruments to allow data cross-referencing.

### Statistical analysis

Prior to analysis, the dataset was cleaned for eligibility, completeness, and consistency of records. Children were excluded if they did not provide all mandatory reports, did not meet the inclusion criteria (i.e., age), or presented inconsistent fruit intake records. These included atypical high consumption values observed in isolated assessment periods, likely arising from misinterpretation of the reporting instructions, whereby individual fruit items (e.g., strawberries) were counted instead of standardized fruit portions. The number of participants excluded for each reason is shown in the results section.

Only children with sociodemographic characterization and both baseline and post-intervention data were included in the analysis (per protocol approach). Behavioral changes, focused on regular fruit intake at school, were assessed using the subsample of children who completed the 12-week classroom tracking chart every day.

Regular fruit intake was defined as eating fruit on at least 75% of school days per week (i.e., four out of five school days, or three out of four in weeks with a holiday), sustained for a minimum of four consecutive weeks and maintained until the end of the 12-week intervention.

Data distribution was assessed with the Kolmogorov-Smirnov test. Categorical variables were presented as frequencies and continuous variables as mean (standard deviation) or median (interquartile range: 25th−75th percentile), according to data distribution. Baseline differences between the total sample and subsample were explored using Fisher’s exact test (2 × 2 nominal data), Chi-square (categorical variables), and the Mann-Whitney U (continuous variables).

The adequacy of the ‘*Fruit Heroes*^®’^ intervention was assessed by comparing overall fruit intake before and after the intervention. Differences in the number of fruit portions consumed by each child were analyzed using Student’s t-test for paired samples, while between-group differences were examined with the Chi-square test or Student’s t-test, as appropriate. McNemar’s test was applied for non-parametric data. To contextualize the magnitude of the observed effect, changes in overall fruit intake were compared with a meta-analysis of school-based interventions, which reported a mean increase of 0.24 portions/day [16].

Data from the classroom tracking chart were used to evaluate regular fruit intake at school (i.e., during morning/afternoon snacks, recorded on a daily basis) and to assess changes in consumption patterns over time. This analysis was limited to children not meeting fruit intake recommendations at baseline (< 3 portions/day).

Logistic regression was used to estimate associations between baseline characteristics (sex, age, and participation status) and adherence to Portuguese fruit intake recommendations (3–5 portions/day), with results reported as odds ratio (OR) and adjusted odds ratio (aOR), with corresponding 95% confidence intervals (95% CI).

Kaplan–Meier survival curves were generated to assess the timing of enacting the regular fruit intake behavior at school, and log-rank tests were used to compare groups (children previously exposed to the intervention vs. first-time participants). The event was defined as the enactment of regular fruit intake, while children who did not achieve this outcome by the end of the follow-up period were censored. Time was defined as the week this behavior was first established. A multivariate Cox regression model was applied to calculate unadjusted and adjusted hazard ratios (HR and aHR, respectively) for the association between enactment of a regular fruit intake and sex, age, and participation status.

Given the clustered structure of the data (children in classrooms), both logistic and Cox regression analyses were performed using the complex sampling procedure, specifying classroom as the sampling unit (cluster) to account for the design effect.

All analyses were conducted using IBM SPSS^®^ Statistics for Macintosh, version 29.0 (2025, Armonk, NY: IBM Corp). Statistical significance was set at *p* < 0.05 (two-sided).

## Results

**Fig. 1:**
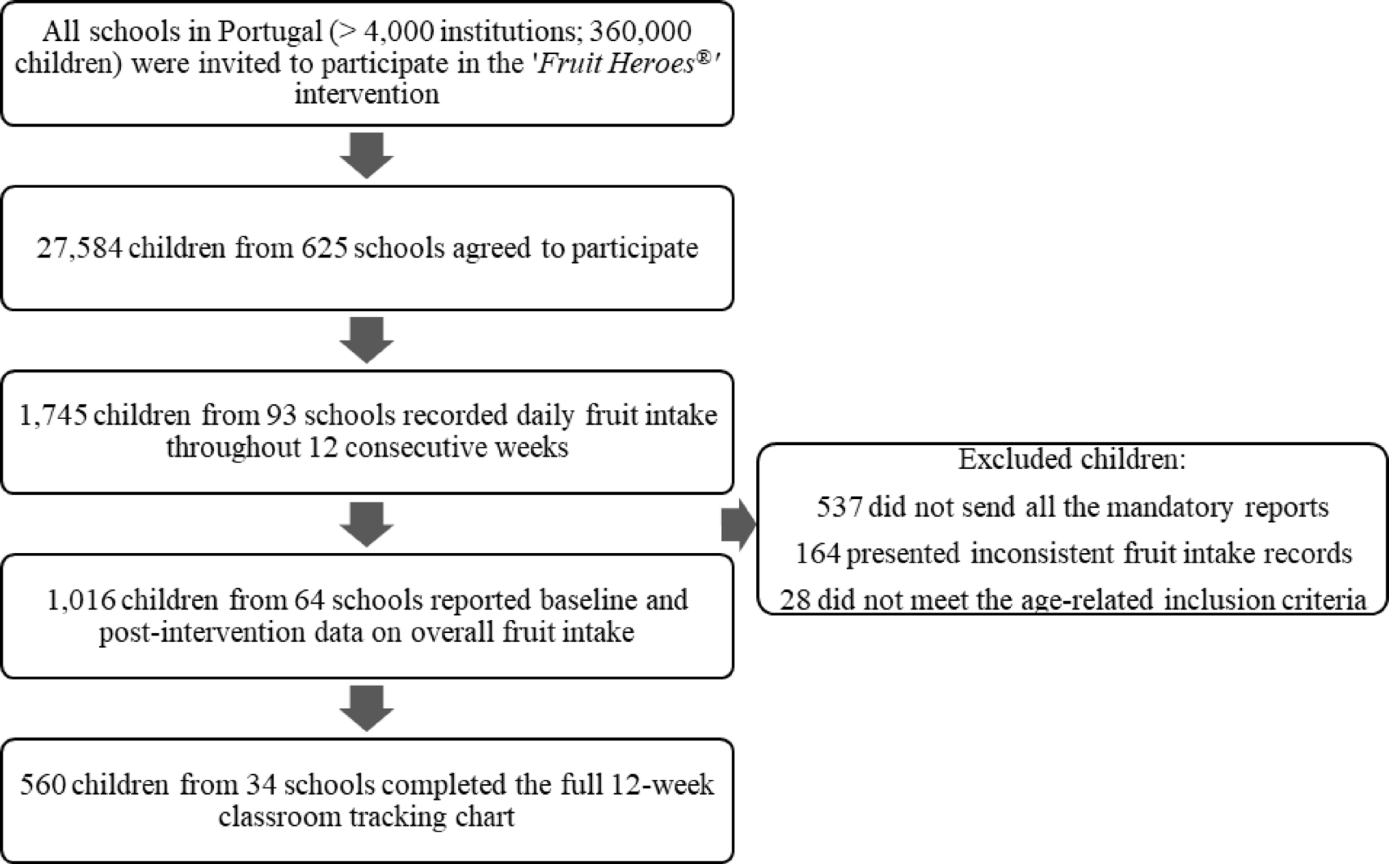
Schools and children participating in the 2018–2019 edition of the ‘*Fruit Heroes*^®^’ project: study flowchart

The number of children (and schools) included in the ‘*Fruit Heroes*^®’^ intervention and its evaluation is summarized in Fig. 1. Around 360,000 children were invited to participate, of whom 27,584 from 625 schools agreed to take part. The present study was based on a subset of participants who provided detailed fruit intake monitoring data. Specifically, 1,745 children (6.3% of the sample who agreed to participate; 93 schools) recorded daily fruit intake over 12 consecutive weeks. After data cleaning, 1,016 children (58.2%; 64 schools) were included in the analysis of overall fruit intake, having provided both baseline and post-intervention data on the number of fruit portions consumed on the previous day. Within this group, 560 children (32.1% of the sample who agreed to participate; 34 schools) completed the full 12-week daily tracking chart and were considered for the assessment of regular fruit intake.

Table 1 presents the baseline characteristics of the total sample (*N* = 1016) and the subsample of children who completed the 12-week daily fruit intake report (*n* = 560). The median age of the total sample was 6.00 years [IQR 4.00–8.00], while the subsample’s was slightly higher, at 7.00 years [IQR 5.00–8.00; *p* < 0.001]. Overall, 68.8% of the total sample consumed fewer than three daily portions at baseline. However, the percentage of children meeting national recommendations was significantly higher among those who completed the 12-week reporting (35.4% vs. 30.7%; *p* < 0.001).

**Table 1:** Baseline characteristics of the total sample and the subsample of children completing 12 weeks of fruit intake reporting

	Total sample (*N* = 1016)	Subsample (*n* = 560)	*p*-value^*,**^
*Age group, years*	6.00 [4.00–8.00]	7.00 [5.00–8.00]	< 0.001
3–5 years	446 (43.9)	193 (34.5)^b^	**< 0.001**
6–8 years	415 (40.8)	263 (47.0)^a^	
9–11 years	155 (15.3)	104 (18.6)^a^	
*Sex*			
Girls	521 (51.3)	288 (51.4)	0.950
Boys	495 (48.7)	272 (48.6)	
*Participation status in the* *Fruit Heroes*^®^			
First-time	462 (45.5)	254 (45.4)	0.950
Previous exposure	554 (54.5)	306 (54.6)	
*Baseline fruit intake* ^†^			
Below recommendations (< 3 portions/day)	699 (68.8)	357 (63.8)	**< 0.001**
Within recommendations (3–5 portions/day)	312 (30.7)	198 (35.4)	
Above recommendations (> 5 portions/day)	5 (0.5)	5 (0.9)	

Data are presented as median [IQR] or n (%). Bold indicates statistical significance (*p* < 0.05)

^*^Comparison between participants who reported daily fruit consumption throughout the 12 weeks of the intervention with those who did not

^**^Fisher’s exact test for 2 × 2 nominal data, chi-square test for categorical data, and Mann-Whitney U for continuous data

^a^Adjusted standardized residuals > 1.96, indicating that the subcategory was observed more frequently than expected if the variables were independent

^b^Adjusted standardized residuals < − 1.96, indicating that the subcategory was observed less frequently than expected

^†^Portuguese Food Wheel Guide criteria

The *p-value* for baseline fruit intake excludes participants in the “above recommendations” category due to the small sample size (*n* = 5)

In complex samples logistic regression models accounting for clustering at the classroom level and controlling for sex and age group, children previously exposed to the *‘Fruit Heroes*^®’^ had significantly higher odds of meeting fruit intake recommendations at baseline compared with first-time participants (aOR = 2.24; 95%CI 1.27–3.94; Table 2).

**Table 2:** Association between baseline characteristics and meeting fruit intake daily recommendations, according to the Portuguese Food Wheel Guide (*N* = 1016)

	< 3 daily portions (*n* = 699)^†^	3–5 daily portions (*n* = 312)^†^	> 5 daily portions (*n* = 5)^†^	Crude OR (95% CI)	Adjusted OR (95% CI)^a^
*Sex*					
Girls	353 (50.5)	141 (45.2)	1 (20.0)	1	1
Boys	346 (49.5)	171 (54.8)	4 (80.0)	1.26 (0.96–1.65)	1.25 (0.96–1.63)
*Age group*					
3–5 years	342 (48.9)	103 (33.0)	1 (20.0)	1	1
6–8 years	268 (38.3)	143 (45.8)	4 (80.0)	1.35 (0.66–2.76)	1.23 (0.65–2.39)
9–11 years	89 (12.7)	66 (21.2)	0 (0.0)	**2.44 (1.05–5.67)**	2.23 (0.99–4.99)
*Participation status in the Fruit Heroes* ^®^					
First-time	362 (51.8)	99 (31.7)	1 (20.0)	1	1
Previous exposure	337 (48.2)	213 (68.3)	4 (80.0)	**2.33 (1.32–4.11)**	**2.24 (1.27–3.94)**

OR, Odds Ratio; CI, Confidence Interval. Data are presented as n (%) or OR (95% CI). Bold indicates statistical significance (*p* < 0.05)

^†^Portuguese Food Wheel Guide criteria

^a^Adjusted for sex, age group, and participation status

OR estimates, both crude and adjusted, result from complex samples logistic regression models accounting for clustering at the classroom level

Mean overall fruit intake increased from 2.09 (1.12) to 2.44 (1.02) daily portions after the intervention (*p* < 0.001). A significant rise was also observed in the percentage of children meeting recommendations (from 30.7% to 44.4%, *p* < 0.001).

**Fig. 2:**
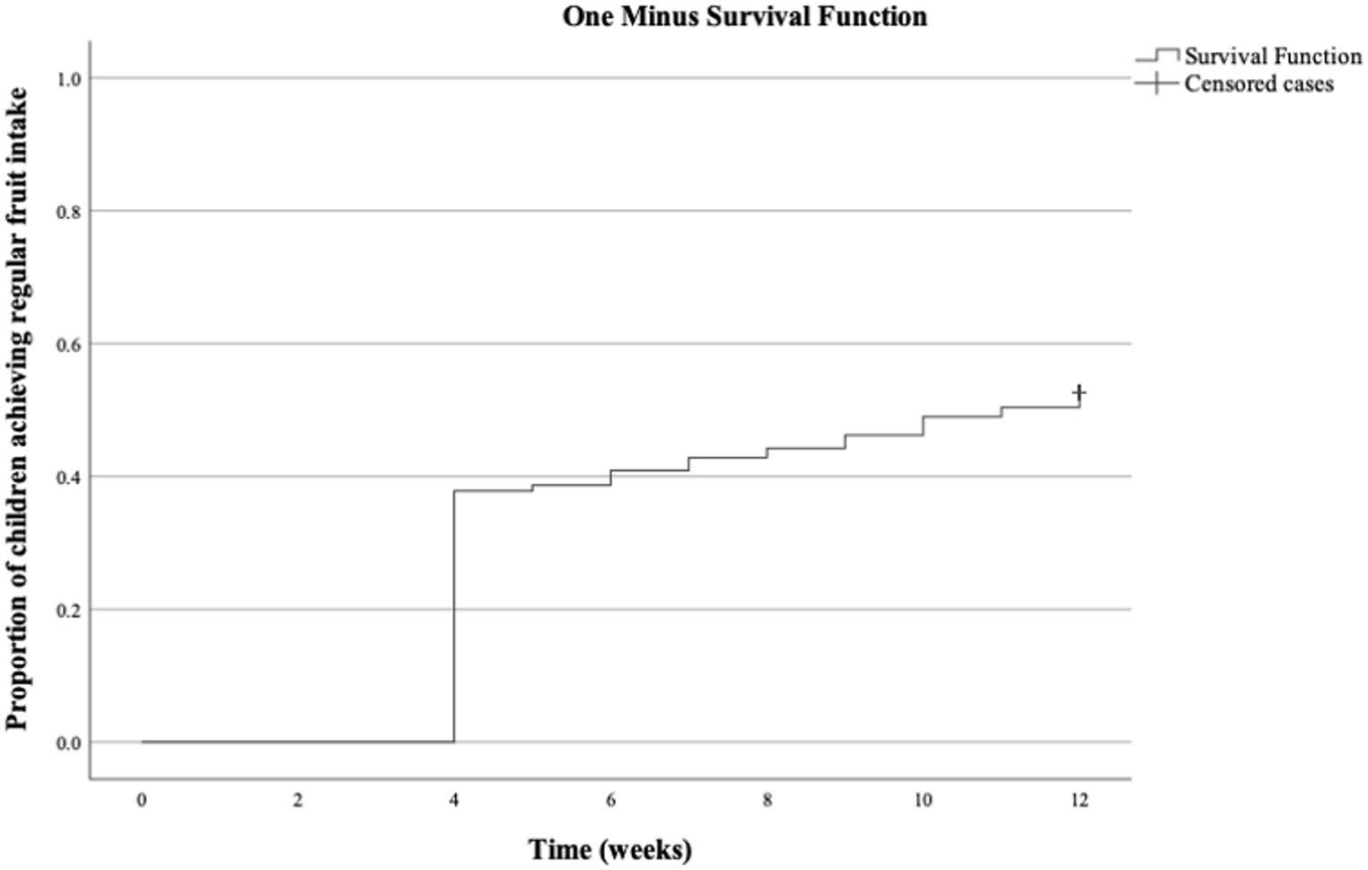
Kaplan-Meier survival curve showing the proportion of children achieving regular fruit intake over the 12-week intervention period, for the subsample (*n* = 357); censored observations (i.e., participants who did not achieve regular fruit intake) are indicated by (+) mark

Figure 2 illustrates the weekly evolution in the proportion of children enacting regular fruit intake, among the subsample who provided daily records and began the intervention with suboptimal intake (*n* = 357). At completion, 52.7% had established regular fruit intake at school. After the first four weeks, 37.8% of the children had already achieved the behavior and maintained it until the end of the intervention.

The weekly number of new children achieving regular fruit intake and maintaining it until the end of the intervention ranged from 3 to 10 (weeks 5 and 10, respectively). The median time for half of the children to establish regular intake was 11.70 weeks (Supplementary Table S1). Survival estimates stratified by participation status (previous exposure vs. first-time) are presented in Supplementary Table S2.

**Fig. 3:**
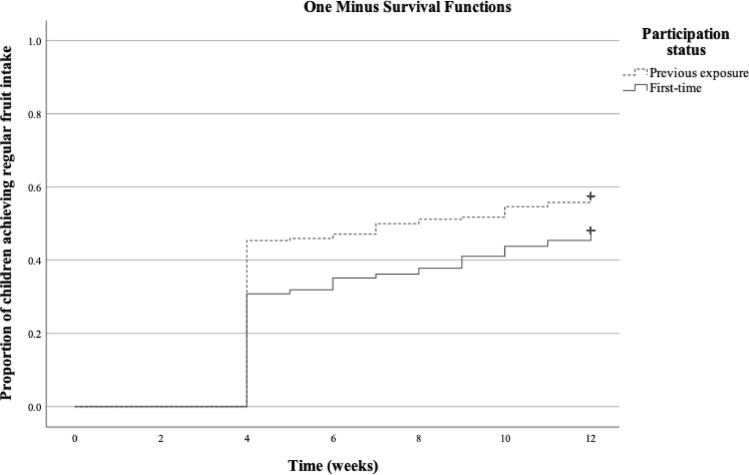
Kaplan-Meier survival curves showing the proportion of children achieving regular fruit intake over the 12-week intervention period, stratified by participation status (previous exposure vs. first-time; *n* = 357); censored observations (i.e., participants who did not achieve regular fruit intake) are indicated by (+) mark

Children previously exposed to the intervention performed better in enacting regular fruit intake than first-time participants, although the difference did not reach statistical significance (Fig. 3; *p* = 0.051). By week 4, regular intake was more frequent among recurrent participants (45.3%) compared to new ones (30.8%).

Crude hazard ratios indicated a positive association between age group and the likelihood of enacting regular fruit intake (Table 3). In the adjusted model, accounting for clustering at the classroom level and controlling for sex, age group, participation status, and baseline fruit intake, children aged 6–8 years had a significantly higher probability of consuming fruit regularly at school (aHR = 2.24; 95%CI 1.29–3.88), compared with those aged 3–5 years. Although children previously exposed to the intervention showed a higher likelihood of reaching regular fruit intake, this association was not statistically significant.

**Table 3:** Determinants of regular fruit intake at school (proxy for habit formation): Cox proportional hazard models (*n* = 357)

	Regular fruit intake	Crude HR (95%CI)	Adjusted HR (95%CI)^a^
*Sex*			
Girls	102 (57.0)	1	1
Boys	86 (48.3)	1.28 (0.89–1.85)	1.34 (0.95–1.89)
*Age group, years*			
3–5 years	51 (37.8)	1	1
6–8 years	100 (64.1)	**2.26 (1.30**–**3.93)**	**2.24 (1.29**–**3.88)**
9–11 years	37 (56.1)	1.85 (0.70–4.86)	1.85 (0.69–4.91)
*Participation in the Fruit Heroes* ^®^			
First-time	89 (48.1)	1	1
Previous exposure	99 (57.6)	1.37 (0.76–2.46)	1.27 (0.72–2.24)

HR, Hazard Ratio; CI, Confidence Interval. Data are presented as n (%) or HR (95%CI). Bold indicates statistical significance (*p* < 0.05)

^a^Adjusted for sex, age group, participation status, and baseline fruit consumption according to national dietary recommendations

^†^Portuguese Food Wheel Guide criteria

HR estimates, both crude and adjusted, result from Complex Samples Cox regression models accounting for clustering classroom at the classroom level

## Discussion

This study assessed the adequacy of a 12-week school-based intervention –*‘Fruit Heroes*^®’^– in promoting and sustaining fruit intake among pre- and primary-school children (3–11 years) in Portugal, using a before-and-after design.

At baseline, no sociodemographic differences were found between the total and the subsample who reported daily fruit intake, except for age. Regarding fruit intake, a higher proportion of children in the subsample met the national recommendations (35.4% vs. 30.7%).

Compared with other research that reported 60% of children meeting fruit intake recommendations at baseline [13], we observed a lower proportion (30.7%). This discrepancy likely reflects differences in dietary guidelines: the Portuguese recommendations define one fruit portion as 160 g and set a minimum of 480 g per day (three portions) [7], more than twice the 200 g (two portions) threshold used by De Bock and colleagues [13].

Children’s overall fruit intake improved by the end of the intervention. Both the mean number of fruit portions consumed on the previous day and the percentage of participants meeting the national recommendation of 3–5 daily portions significantly increased after the 12-week initiative. At baseline, a higher proportion of children previously exposed to the intervention met fruit recommendations. Among the subsample who reported daily intake throughout the 12 weeks (tracking school chart), more than half achieved regular fruit intake, particularly children aged 6–8 years. These findings suggest that ‘*Fruit Heroes*^®’^ may foster the adoption of regular fruit intake and that repeated exposure to it may progressively reinforce healthy eating behaviors.

Our results are consistent with previous studies reporting the effectiveness of school-based interventions that also used mascot/cartoon characters and reward systems to encourage fruit intake in children, both in pre-post analyses [21] and controlled trials [22].

In this study, overall fruit intake increased by 0.35 portions per day—exceeding the 0.24-portion rise reported in a meta-analysis of 21 school-based interventions lasting three to nine months [16]. These comparisons should, however, be interpreted cautiously, as the present study lacked a control group, unlike those included in the meta-analysis. A controlled study conducted in Portugal also reported a positive impact of a school-based intervention on improving fruit intake, particularly when served as dessert [14]. It is plausible to hypothesize that the results observed in our study were influenced by the cultural context in which the intervention was developed—Portugal, a southern European country embedded in the Mediterranean dietary tradition [23], characterized by an abundance of plant-based foods such as fruits and vegetables. However, adherence to this dietary pattern among Portuguese children and adolescents remains low [24, 25].

An increase of 0.35 fruit portions per day may translate into meaningful population-level benefits. Since 2.4% of disability-adjusted life years and 4.0% of deaths worldwide are attributable to low fruit and vegetable intake [26], any gain in fruit intake can potentially reduce these outcomes [27]. Moreover, the proportion of children meeting Portuguese fruit recommendations increased by approximately 45% after the intervention—an encouraging finding considering the nationwide implementation of the *‘Fruit Heroes*^®’^ intervention.

Studies assessing the long-term effectiveness of fruit and vegetable promotion initiatives have shown that effects tend to weaken over time [28]. Because *‘Fruit Heroes*^®’^ is an annual intervention, it is particularly relevant to compare fruit intake between first-time participants and those previously exposed to the initiative. The findings suggest that prior participation may positively influence fruit intake: (1) at baseline, children with previous exposure had a 2.24-fold of meeting the fruit recommendations; and (2) survival analysis indicated that recurrent participants achieved regular fruit intake at school more frequently and earlier than first-time participants. These results suggest that the intervention may maintain its adequacy for at least one year, or that children with previous exposure are more engaged with this gamified initiative. It is also plausible that participation in earlier editions fostered greater awareness or preference for fruit, as reported in other studies [21]. These hypotheses are of great interest for future exploration and are encouraging for their potential long-term health implications, since dietary habits established in childhood tend to persist into adulthood [2]. Future evaluations of the initiative should include baseline, post-intervention, and follow-up assessments to assess sustainability (at least six months after the end of the intervention).

More than half of the participating children completed the intervention. Among those who reported daily fruit intake over the 12 weeks and started the intervention consuming fewer than three portions per day, survival analysis was conducted to estimate the weekly incidence of children achieving regular fruit intake (i.e., consuming fruit on at least 75% of school days for four consecutive weeks and maintaining the behavior until week 12). To our knowledge, this is the first study to apply survival analysis to assess the adoption of an eating habit. The definition of ‘regular fruit intake’ was specifically developed by the research team for this purpose. The 75% threshold corresponds to approximately four out of five school days (or three out of four), reflecting consistent behavior while accommodating variations in school attendance – national holidays or strikes that occurred during the intervention period. The minimum duration of four consecutive weeks was informed by [18], who reported that the lower bound for the time required for a behaviour to reach 95% of its automaticity asymptote was 18 days. Our results showed that more than half of the children in the subsample reached regular fruit intake by week 12. Notably, 37.8% of participants had already presented this behavior after the fourth week (considering a *per-protocol* approach, i.e., including only children who have completed 12 weeks of follow-up). Based on these findings, the intervention duration was shortened from 12 to five weeks in subsequent editions to enhance feasibility and engagement, while reducing the workload associated with extended implementation for teachers.

The intervention appeared particularly suitable for promoting regular fruit intake among children previously exposed. Stratified analyses showed that differences between groups were mainly attributable to the first four weeks, as no relevant changes were observed beyond week five. Two possible explanations emerge: (1) children previously exposed to the intervention already presented regular fruit intake before being exposed for the first time (potential learning effect); or (2) repeated exposure enhances responsiveness (reinforcement effect). The use of repeated exposure as a behavior-change strategy aligns with the Theory of Mere Exposure [29], which predicts that repeated exposure to specific stimuli—including healthy foods—increases the likelihood of future engagement [30, 31]. Regarding the association with age, since ‘*Fruit Heroes*^®’^ is implemented annually, recurrent participants are typically older, and age is positively correlated with fruit intake, as described by other authors [32].

Evaluating initiatives such as *‘Fruit Heroes*^®’^ is essential to understand their effectiveness in fostering healthy habits in schools and for highlighting the potential benefits of structured nutrition curricula—currently missing in Portugal, especially for younger children.

### Strengths and limitations

*‘Fruit Heroes*^®’^ was originally designed as a community-based initiative rather than a research project. Although it provides valuable real-world data that enables assessment of its adequacy, several methodological limitations should be acknowledged.

This study included over 1000 children from all regions of Portugal, within an intervention that reached more than 25,000 participants nationwide. Using a convenience sample increased feasibility and allowed the inclusion of a large number of participants. Yet, this approach introduces potential selection bias, as schools involved in previous editions were likely more motivated to participate again.

Encouraging fruit intake during morning/afternoon snacks is a relevant strategy; however, information on other meals was not collected, limiting the ability to assess whether increased fruit intake at snacks translated into higher overall consumption or displacement of foods high in fat, sugar, or salt. Moreover, since regular fruit intake at school was not measured before the intervention, it was not possible to distinguish between newly acquired and pre-existing habits. As such, the analysis of regular intake throughout the intervention relied on survival models rather than pre–post comparisons. Additionally, the study lacked comprehensive information on potentially relevant covariates, including socioeconomic status, body mass index, and maternal age. These variables are well-established predictors of children’s dietary behaviours and may act as important confounders in the relationship between intervention exposure and fruit intake. However, due to their incomplete or inconsistent availability in the dataset, it was not possible to include them in the adjusted analyses. This limitation should be considered when interpreting the observed associations. Future evaluations should incorporate these covariates to better characterise heterogeneity in intervention response and to strengthen causal inference regarding changes in dietary behaviour. Despite this limitation, the large and geographically diverse sample provides valuable real-world evidence on the potential effectiveness of the intervention.

Children’s daily reports (classroom tracking chart) captured fruit intake during morning and afternoon snacks. Using a quantitative (or polytomous) scale instead of a yes/no format would allow a clearer understanding of which meal contributed most to improvements in consumption. However, given the young age of the participants, a simpler format was used, limiting the depth of the collected information.

Support materials were provided to clarify portion sizes, although inaccuracies may have arisen from children’s recall of fruit intake or teachers’ conversion of reported items into portions.

The absence of a control group restricts causal inference. This limitation is currently being addressed through a two-arm cluster randomized controlled trial, with schools as the unit of randomization to the intervention or control groups. A follow-up is planned to assess fruit intake at six months post-intervention.

Furthermore, teachers’ attitudes and beliefs regarding the role of schools in health promotion—beyond traditional educational (e.g., mathematics and other subjects)—are also being assessed in the ongoing national evaluation of the intervention.

## Conclusions

This study demonstrates the adequacy of the *‘Fruit Heroes*^®’^ school-based intervention in improving children’s fruit intake. Repeated exposure to these initiatives may have a role in consolidating healthy eating habits early in life and, therefore, be sustainable until adolescence and adulthood, generating long-term health benefits.

The findings reinforce the importance of school-based interventions and the role of teachers in fostering healthy habits, especially when integrating gamification and storytelling in their pedagogic toolboxes. However, achieving sustainable changes in children’s dietary habits requires a systemic approach—integrating school, family, and social environments, and actively involving parents and caregivers in nutrition education.

## Supplementary Information

Below is the link to the electronic supplementary material.

Supplementary Material 1

## Data Availability

The anonymized dataset is available on Zenodo (10.5281/zenodo.17482981).
