# Supplementary material for: Promoting and sustaining fruit intake among children aged 3–11 years: a before-and-after evaluation of a school-based intervention
Source: Eur J Nutr. 2026 Jun 29;65(5):184. doi: 10.1007/s00394-026-04042-3 (PMC13314690; doi:10.1007/s00394-026-04042-3)
Supplement: Supplementary file 1 — Supplementary Material 1 [file 394_2026_4042_MOESM1_ESM.pdf]

## Promoting and sustaining fruit intake among children aged 3–11 years: A before-and-after evaluation of a school-based intervention

## Supplementary Material

**Supplementary Table 1** Survival estimates regarding regular fruit intake (at morning or afternoon snacks) among participants reporting daily consumption throughout the 12-week intervention (n=357)

**Life Table[a]**

| Interval Start Time (week) | Number Entering Interval | Number Withdrawing during Interval | Number Exposed to Risk | Number of Terminal Events | Proportion Terminating | Proportion Surviving | Cumulative Proportion Surviving at End of Interval | Std. Error of Cumulative Proportion Surviving at End of Interval | Probability Density | Std. Error of Probability Density | Hazard Rate | Std. Error of Hazard Rate |
|---|---|---|---|---|---|---|---|---|---|---|---|---|
| 0 | 357 | 0 | 357.000 | 0 | .00 | 1.00 | 1.00 | .00 | .000 | .000 | .00 | .00 |
| 1 | 357 | 0 | 357.000 | 0 | .00 | 1.00 | 1.00 | .00 | .000 | .000 | .00 | .00 |
| 2 | 357 | 0 | 357.000 | 0 | .00 | 1.00 | 1.00 | .00 | .000 | .000 | .00 | .00 |
| 3 | 357 | 0 | 357.000 | 0 | .00 | 1.00 | 1.00 | .00 | .000 | .000 | .00 | .00 |
| 4 | 357 | 0 | 357.000 | 135 | .38 | .62 | .62 | .03 | .378 | .026 | .47 | .04 |
| 5 | 222 | 0 | 222.000 | 3 | .01 | .99 | .61 | .03 | .008 | .005 | .01 | .01 |
| 6 | 219 | 0 | 219.000 | 8 | .04 | .96 | .59 | .03 | .022 | .008 | .04 | .01 |
| 7 | 211 | 0 | 211.000 | 7 | .03 | .97 | .57 | .03 | .020 | .007 | .03 | .01 |
| 8 | 204 | 0 | 204.000 | 5 | .02 | .98 | .56 | .03 | .014 | .006 | .02 | .01 |
| 9 | 199 | 0 | 199.000 | 7 | .04 | .96 | .54 | .03 | .020 | .007 | .04 | .01 |
| 10 | 192 | 0 | 192.000 | 10 | .05 | .95 | .51 | .03 | .028 | .009 | .05 | .02 |
| 11 | 182 | 0 | 182.000 | 5 | .03 | .97 | .50 | .03 | .014 | .006 | .03 | .01 |
| 12 | 177 | 169 | 92.500 | 8 | .09 | .91 | .45 | .03 | .000 | .000 | .00 | .00 |

a. The median survival time is 11.70

**Supplementary Table 2** Survival estimates regarding regular fruit intake (at morning or afternoon snacks) among participants reporting daily consumption throughout the 12-week intervention (n=357), stratified by participation status (previous exposure *vs.* first-time)

**Life Table**

| First-order Controls | Interval Start Time (weeks) | Number Entering Interval | Number Withdrawing during Interval | Number Exposed to Risk | Number of Terminal Events | Proportion Terminating | Proportion Surviving | Cumulative Proportion Surviving at End of Interval | Std. Error of Cumulative Proportion Surviving at End of Interval | Probability Density | Std. Error of Probability Density | Hazard Rate | Std. Error of Hazard Rate |
|---|---|---|---|---|---|---|---|---|---|---|---|---|---|
| **Previously exposure participants** | 0 | 172 | 0 | 172.000 | 0 | .00 | 1.00 | 1.00 | .00 | .000 | .000 | .00 | .00 |
| | 1 | 172 | 0 | 172.000 | 0 | .00 | 1.00 | 1.00 | .00 | .000 | .000 | .00 | .00 |
| | 2 | 172 | 0 | 172.000 | 0 | .00 | 1.00 | 1.00 | .00 | .000 | .000 | .00 | .00 |
| | 3 | 172 | 0 | 172.000 | 0 | .00 | 1.00 | 1.00 | .00 | .000 | .000 | .00 | .00 |
| | 4 | 172 | 0 | 172.000 | 78 | .45 | .55 | .55 | .04 | .453 | .038 | .59 | .06 |
| | 5 | 94 | 0 | 94.000 | 1 | .01 | .99 | .54 | .04 | .006 | .006 | .01 | .01 |
| | 6 | 93 | 0 | 93.000 | 2 | .02 | .98 | .53 | .04 | .012 | .008 | .02 | .02 |
| | 7 | 91 | 0 | 91.000 | 5 | .05 | .95 | .50 | .04 | .029 | .013 | .06 | .03 |
| | 8 | 86 | 0 | 86.000 | 2 | .02 | .98 | .49 | .04 | .012 | .008 | .02 | .02 |
| | 9 | 84 | 0 | 84.000 | 1 | .01 | .99 | .48 | .04 | .006 | .006 | .01 | .01 |
| | 10 | 83 | 0 | 83.000 | 5 | .06 | .94 | .45 | .04 | .029 | .013 | .06 | .03 |
| | 11 | 78 | 0 | 78.000 | 2 | .03 | .97 | .44 | .04 | .012 | .008 | .03 | .02 |
| | 12 | 76 | 73 | 39.500 | 3 | .08 | .92 | .41 | .04 | .000 | .000 | .00 | .00 |
| **First-time participants** | 0 | 185 | 0 | 185.000 | 0 | .00 | 1.00 | 1.00 | .00 | .000 | .000 | .00 | .00 |
| | 1 | 185 | 0 | 185.000 | 0 | .00 | 1.00 | 1.00 | .00 | .000 | .000 | .00 | .00 |
| | 2 | 185 | 0 | 185.000 | 0 | .00 | 1.00 | 1.00 | .00 | .000 | .000 | .00 | .00 |
| | 3 | 185 | 0 | 185.000 | 0 | .00 | 1.00 | 1.00 | .00 | .000 | .000 | .00 | .00 |
| | 4 | 185 | 0 | 185.000 | 57 | .31 | .69 | .69 | .03 | .308 | .034 | .36 | .05 |
| | 5 | 128 | 0 | 128.000 | 2 | .02 | .98 | .68 | .03 | .011 | .008 | .02 | .01 |
| | 6 | 126 | 0 | 126.000 | 6 | .05 | .95 | .65 | .04 | .032 | .013 | .05 | .02 |
| | 7 | 120 | 0 | 120.000 | 2 | .02 | .98 | .64 | .04 | .011 | .008 | .02 | .01 |
| | 8 | 118 | 0 | 118.000 | 3 | .03 | .97 | .62 | .04 | .016 | .009 | .03 | .01 |
| | 9 | 115 | 0 | 115.000 | 6 | .05 | .95 | .59 | .04 | .032 | .013 | .05 | .02 |
| | 10 | 109 | 0 | 109.000 | 5 | .05 | .95 | .56 | .04 | .027 | .012 | .05 | .02 |
| | 11 | 104 | 0 | 104.000 | 3 | .03 | .97 | .55 | .04 | .016 | .009 | .03 | .02 |
| | 12 | 101 | 96 | 53.000 | 5 | .09 | .91 | .49 | .04 | .000 | .000 | .00 | .00 |
